# Supplementary material for: Vaccine uptake, associated factors and reasons for vaccination status among the South African elderly; findings and next steps
Source: PLoS One. 2024 Dec 4;19(12):e0314098. doi: 10.1371/journal.pone.0314098 (PMC11616853; doi:10.1371/journal.pone.0314098)
Supplement: Attachment — Submitted filename: Response to Reviewers.docx [file pone.0314098.s002.docx]

**Emily Chenette**

**Editor-in-Chief: PLOS ONE**

**1265 Battery Street, Suite 200**

**San Francisco, CA 94111**

**United States**

Dear Dr Chenette,

**RE: RESUBMISSION OF MANUSCRIPT: *Vaccine uptake, associated factors and reasons for vaccination status among the South African elderly; findings and next steps***

I hope this letter finds you well. I am writing in response to the reviewers' comments on the above-mentioned manuscript.

We are grateful to the reviewers for their time and thoughtful feedback on our work. We have carefully considered each of the points raised, addressed them in the manuscript and provide details, including line refences, below.

**Reviewer #1**

1. The authors performed a multivariate analysis to identify factors associated with uptake of influenza and pneumococcal vaccination. In the abstract, the authors identify significant predictors of vaccine uptake, such as residing in the Western Cape Province, but do not address the same predictors in the results section, except for in Table 3. The authors should describe in more details the significant predictors they identified.

Response: We thank you for this comment. We had not reported factors in the text part of the results section that were reported in the Table, in order to avoid repetition of the results. We have rectified this by reporting factors in the text with their odds ratios, and in the table with odds ratios and 95% CIs (lines 248-256). A description of the significant predictors / barriers is also provided in the discussion (lines 283-291). We hope this is acceptable.

2. The authors should provide more context on why different residing in different provinces, race and education may contribute to increased odds of receiving a vaccine. The factors were highlighted in the abstract and reported in Table 3 but were not contextualized in the discussion section.

Response: Thank you for this comment. The context for socio-demographic factors identified in our study is now provided in the discussion section (lines 283-291), and we trust this is now OK.

3. The authors recommend extending free access to other vaccines, including the pneumococcal vaccine. The authors should define what “other vaccines” they believe should be free.

Response: Thanks for this comment, which has been addressed by naming both pneumococcal and shingles vaccines (see line 486-487).

Additionally, their own data does not seem support their recommendation where they report that only 0.9% and 1.4% of individuals who did not receive the pneumococcal and shingles vaccine identified cost as a major obstacle. The authors should support their recommendations with data from their own study and/or provide more context to why the recommendation should be given considering their own results.

Response: Thanks very much for highlighting this, as we had omitted the cost obstacle results for those who were aware of these vaccines. We have now addressed this in lines 234-236. We trust this is now clear.

4. In the recommendation section the authors recommend the use of health technologies such as electronic medical records. The authors should provide the reason for this recommendation.

Response: Thank you, an explanation for this is now provided in the recommendations (lines 499-505).

5. The authors highlight a feature of the South African COVID-19 vaccination program where vaccine services were brought to the elderly. The authors should provide citations for the program.

Response: Thank you for this comment. A citation has now been included (Ref 110).

**Reviewer #2**

1. Please describe the efficacy of the three types of vaccines (influenza, pneumococcal, and shingles) studied.

Response: Thank you, this information is now added to the relevant parts of the discussion section of the manuscript (lines 344-346, 382-386 and 422-425).

2. Please discuss why the rate of influenza vaccination is so low in Shanghai and so high in Brazil.

Response: This has now been added in the discussion (lines 335-339), and we trust this is now OK.

We believe that these revisions strengthen the manuscript and address the concerns raised by the reviewers. Thank you once again for the opportunity to submit our work to your Journal We appreciate your consideration of our response to the review and look forward to hearing from you soon.

Yours sincerely,

_________________________

**Mr Mncengeli Sibanda**
3 May 2024
